# Process optimization in poultry feed mill

**DOI:** 10.1038/s41598-023-36072-w

**Published:** 2023-06-19

**Authors:** Sameh A. Salaheldein Amin, Nahed Sobhi

**Affiliations:** 1 Department of Industrial System Engineering, University of October for Modern Science and Arts, Cairo, Egypt; 2 Faculty of Engineering, University of October for Modern Science and Arts, Cairo, Egypt

**Keywords:** Engineering, Mathematics and computing

## Abstract

The poultry feed industry is pretty much active in a lot of countries and it is achieved market acceptance. The final products are supposed to meet certain specifications to fulfill the nutritional need of animals at different life periods. The final product for poultry is shipped in the form of pelleted feed for the convenience of consumption. One of the major challenges of poultry feed production is the principal complement of equipment necessary for the local production. Imported poultry pellets are quite expensive and unaffordable for many poultry feed industries. Hence, the need to be able to produce poultry feed at lower cost yet achieve the objective of quantity and quality expected of pelleted feeds is critical to the viability of the enterprise. The study aims to investigate the effects of some operating parameters (pressure and temperature of the compounded feed) and die hole size on the pelleting efficiency, throughput capacity, and to optimize the conditions. The improvement approach is conducted by observing the main operating parameters of productivity; statistical analysis is conducted to observe the effect of those parameters on the production rate and the quality of the product. Comparison between parameter levels is done through analysis of variance to determine the significance of the tested parameters. The optimization of parameters was applied with Minitab and designed expert software to determine the best operating conditions. The obtained results showed that the downtime decreased by 77% monthly and productivity increased by 32.5% per hour and the pellet durability index increased by 1.23%. The total sales increased by 6,750,600 LE/Month.

## Introduction

In a competitive market, customers are more likely to deal with a company that delivers their desired specifications and needs. Therefore, the companies focus on improving and optimizing their production processes to be competitive in the market. Improving the process required understanding modern technology which is the crucial key to a successful business. After identifying the root cause of the problem and highlighting the bottleneck then process improvement can be achieved. The implementation of the solutions is connected with the form of weekly experiments, and multiple trials to eliminate variance for every experiment. This allows finding solutions to the problem with productivity without violating the general production constraints that can be done through optimizing equipment, or operating parameters. The feed industry must grow to meet the ever-growing demand by poultry farmers. The feed manufacturing industry faces enormous challenges and the demand for better quality feed is increasing gradually, it is becoming essential to improve the processes in a feed mill to increase the capacity while maintaining the required quality^1^. The production of poultry feed consists of five main work stages: the receiving, grinding, mixing, pellet and warehouse. The pellet process represents the heart of the manufacturing process. Pellet can be generally defined as an extrusion type in which the finely reduced particles of the feed into a compact, easily handled, pellet^2^. But the manipulation of feed pellets in storage, transferring, and transporting seriously affects the number of pellets that reach the feeding sites. The best way to solve this issue is to increase Pellet Durability Index (PDI) by using different settings and different raw materials ingredients in the production process. The main objective of this research is to investigate the effect of die hole size and some operating parameters of pelleting machine including (pressure and temperature) on the pelleting efficiency and throughput capacity for poultry feed production. This is essentially based on previous studies of^3,4^ on the locally pelleting press that indicated the importance of these operating parameters. However, there are different studies of factors affecting pellet durability and pelleting efficiency^5,6^. The study will enable smallholder local pellet producers from poultry feed that is compounded from the various ingredients to produce pellets through the right combination of operating parameters. Factors affecting the pellet quality such as mixing, grinding, pellet techniques, and conditioning process are what to be focused on for quality optimization^7^. Many studies were conducted on a pelleting machine to evaluate and manufacture under certain conditions, the experiments were focused on deterring the effect of different die speeds, different die holes’ diameters, and moisture content of feed mixture^8^. Minitab and designed expert software are considered the simplest ways to perform optimization for the operating conditions of the pelleting machine. It helps in solving some very complex optimization problems by making a few simplifying assumptions^9^. Minitab provides comprehensive tools for manipulating the dataset and moving toward useful solutions. It allows users to visualize patterns and trends easily and find the optimal parameters. This work focuses on increasing the productivity of the poultry feed production line. Defining and analyzing the performance of every stage in the production line and recognizing the main cause of production limitations which summarized a bottleneck in the pelleting machine, and downtime due to the stoppage of pelleting machine. Minitab and designed expert software are used to optimization for the operating conditions of the pelleting machine. Changing the die-holes is used to decrease the downtime of the pelleting machine and improve the pellet quality. The achieved results after applying the optimized parameters showed that the downtime of pelleting machine decreases monthly by 77% and the productivity per hour increased by 32.5% and the pellet durability index increased by 1.23%.

## Background and survey

### Feed manufacturing processes

**Figure 1:**
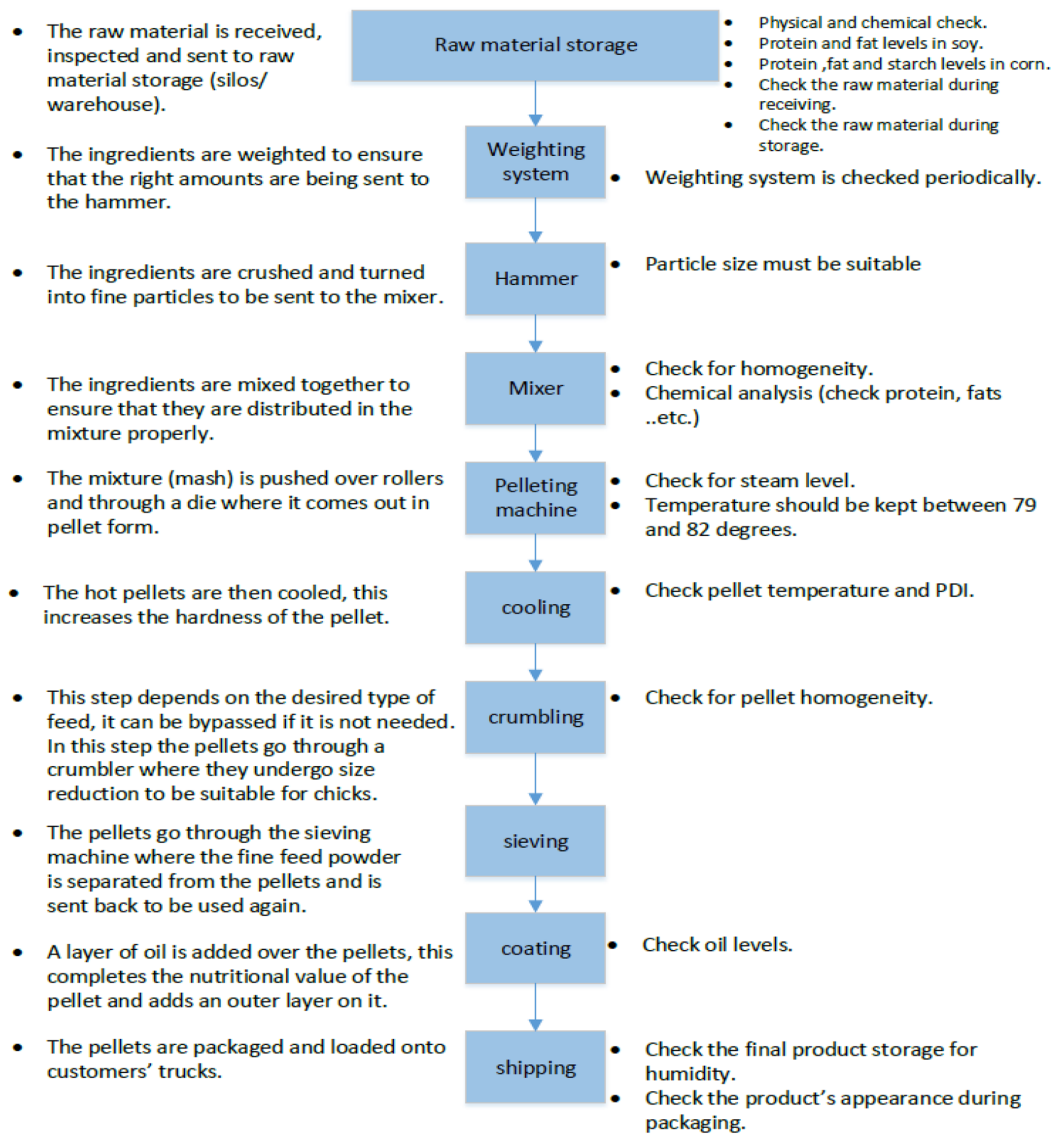
Different processes of feed manufacturing^10^.

Feed undergoes many processes before being packaged and sent off to the farms. The feed steps can be summarized in Fig. 1. The figure also shows the important quality checks to be carried out at each process^10^.

The basic feed manufacturing processes can be summarized in the following steps^10^:

- *Crushing* It is the first step after receiving the raw materials. Any grains go through this process to undergo size reduction and increase the surface area for a greater nutritional value for the poultry^11^. The crushing process is mainly conducted by two types of machines, the hammer mills, and the roller mills:
  *Hammer mills* In hammer mills, particle size reduction is accomplished by impacting slow moving ingredients with a set of hammers moving at high speed. Hammer mills generally produce spherical-shaped particles with the polished surface^10^. The size distribution of particles produced in a hammer mill varies widely around the geometric mean, with some large and many small-sized particles.
  *Roller mills* In roller mills, size reduction is accomplished through a compression force between the rotating roll pairs, producing a homogeneous mixture of ingredients and more uniform particle size distribution with a low proportion of fine materials.
- *Mixing* The main objective of the mixing stage is to start with a certain amount of ingredients having a certain weight and then mix them to an extent where they are homogenous. Any sample taken will have the same amounts of all ingredients. The mixing process can be conducted in many ways; the following are some of the most common methods in the feed industry (horizontal and vertical mixers).
- *Pelleting* it is the process of transforming soft dusty feed into a hard pellet. This process is achieved through compression, extrusion, and adhesion. The process involves passing the feed mixture through a conditioning chamber where steam is added. The moisture from steam provides lubrication for compression and extrusion and in the presence of heat causes some gelatinization of raw starch present on the surface of vegetative ingredients, resulting in adhesion.
- *Cooling* this process aims to reduce the temperature of the pellets resulting from the pelleting process. The cooling of pellets also results in increasing the hardness of the pellet. The cooling process mainly uses vertical coolers or horizontal coolers.
- *Crumbling* this process aims to break down the pellets into small pieces that can be easily consumed by the chicks. It is used when producing starter feed for chicks. Otherwise, it can be bypassed.
- *Sieving* sifting is required when producing pellets. Usually, small fragments (fine) are produced as a result when the hot, moist pellets are cut off from the die inside the pelleting chamber, and as produced pellets pass through the cooling and conveying processes. Fines should be returned to the pelleting machine for reprocessing or can be used as another product which is usually fed to fish.
- *Coating* fats and oils can be added in this process to further improve the nutritional value of the pellets. This aims to add the remaining amount of oils that could not be added before the pelleting process. This process can be done through spraying coaters, vacuum-assisted coaters, or centrifugal coating machines.

### Pelleting process

The pelleting process consists of the following steps^11^:

#### Steam conditioning

Before the pelleting stage steam conditioning of mash is a big step. For the conditioning process to be optimized, a balance between moisture and heat is required. Steam can provide such balance for how easy to control and add, it became quite an important element in the pelleting process. The addition of heat in conditioning is to improve binding and removes any pathogens in the feed.

#### Pelleting

The process of producing pellets is considered a combined mechanical process with moisture, pressure, and heat aggregation of small-sized particles into larger-sized particles^11^. This process is done by passing mash feed from the bin into the conditioner and feeder. Then steam is injected inside the conditioner to the feed, and the conditioned mash flows the pelleting chamber. The formation of pellets occurs by passing the hot mash through the metal die then cooling comes after. The fine partials separated from the pellets can be collected in the sieve and returned into the pellet chamber for reprocessing.

#### Cooling

When pellets leave the chamber, it has a temperature of (80–90) degrees Celsius, carrying moisture between (150 and 170) g/kg. The temperature must be reduced to 8 degrees more than the ambient temperature and moisture should be adjusted between (100 and 120) g/kg. Heat and moisture are removed from pellets by ambient air.

#### Retention time

In the literature, ripening leads to positive effects, with or without steam, and it also points to better-quality pellets. When retention time is more than 5 min more moisture or other liquids can be added followed by steam injection and has no loss effects on productivity or feed quality. However, modern feed manufacturers use a great number of different animal diets that must be produced in short timespans. Therefore, the equipment with a small hold period (less than two minutes) will have a favorable effect on the quality of nutrition.

#### Physical quality of pellets

The quality of the pellet is measured by its ability to withstand abrasion and fragmentation during pneumatic and mechanical movement, storage, or bagging without breaking to reach the feeders without generating a high amount of fines. Pellets are easily fractured from the time they are manufactured until it reaches the chicken, so it creates fines in the feed. The pellet durability test is used to determine the number of solid pellets that remain in manufactured pellets withstanding the attrition stresses due to mechanical process. Pellet durability can be evaluated to estimate the quality of pellets. The quality of the pellet is defined by the PDI^12^.

### Roller and die dimensions

In feed manufacturing, pellet presses use the ring die design. In different designs usually, two or three rollers are used and the die rotates around the centered rollers. A few are designed as flat-die presses in which the die is fixed, and the horizontal rollers revolve while pushing the feed through the die plate. The pressure generated in the die-hole of the pellet press depends on the coefficient of friction between the die wall and feed mash, moisture content, die temperature, the relaxation time of the plastic deformable portion in the mash, and the compressibility of the material^12^.

### Effects of raw material constituents

The constituents include materials such as protein, starch, sugar, fat, non-starch polysaccharides (NSP), fiber, inorganic matter, and water. The matrix structure in which the different components are arranged is very complex and it may lead to preventing the expression of a single constituent on the quality of the pellet^11^. The diet inclusion of fats is a well-known example; free fats that are added into the mixer affect the durability and hardness of the pellet negatively but will improve the capacity of the press in terms of tons produced per hour. This is attributed to the effect of lubricating fat added on the mash-die interface in the pelleting process. A description of the feed constituents’ functionality can be given, this description can vary between different types of grains.

### Pellet mill die and roller design

The pellet dies play a vital role in the pelleting process. Moreover, the type of rollers used to spread and deliver the mash to the die to be formed affects the pelleting process as the following parameters:

#### Die design features

The die physical characteristics are determined by its performance by specifying the overall thickness, correct blank, hole size, and type of relief. The strength of the die is determined by the blank thickness. Resistance to deflection caused by the pelleting process between the die and rolls is increased when the die is thicker. The work of the die material is determined by the effective thickness, therefore affecting pellet quality. If the die material is changed, the effective thickness should be changed as well to maintain good pellet quality and production capacity. To determine the correct (L/d) ratio length of the hole is compared with hole diameter^13^.

#### Die-hole inlets

The hole inlet of the die can be tapered or with an enlarged hole diameter. Tapered inlets act as a chamber for pre-compression, they allow for practical machining techniques to be used and keep the cost of the die lower. Recessed face dies with a high grain ratio, in easy-running applications which are conditioned extremely well and contain a lot of heat and moisture. This type of feed takes the path of the least resistance and will get squeezed between the die and rolls at the outside edge, this prevents the material from escaping and makes sure the die face working width is used effectively. The specifications of the die are identical to the standard die, so the quality of the pellet is the same. When recessing the die face the inside of the die is increased as well as the die’s face area^13^.

#### Die-hole patterns

Three types of patterns are used for die holes: standard, heavy-duty, and close-hole patterns. The standard pattern is suitable for general feed applications with normal hole count, where many formulas are pelleted on the same die and machine. It maintains an average performance but affects the quality and production capacity of the pellets, while also affecting the overall die life. The heavy-duty pattern has less than a normal number of die holes, making the die stronger, and increasing the ligament thickness between the die. Production capacity is reduced, when this pattern is used because of reduced hole count, as well as the cost is increased. The close-hole pattern has several numbers of die-holes more than that of a standard pattern by 25%. By using a closed-hole pattern results in increased pellet quality and production capacity, better die wear, efficient energy use, and the less average cost per ton^14^.

#### Roller shells

Roller shells have many configurations. If maximum performance is expected, the correct design must be chosen accordingly to suit that application. Each design is geometrically engineered to give the highest production of feed through a die while making sure the traction on the die face is reliable. Different designs for different requirements or applications are presented below^15^:

1. *Open-end corrugated shells* They have narrow corrugations and run horizontally over the roller-shell face. It has the most corrugations than any other shell, so it is ideal for any applications that are troubled with chronic roll slip. It is used for manufacturing high-fiber materials such as dairy for cattle feeds^15^.
2. *Closed-end corrugated shells* The shape of its corrugation is gently sloping and closed off at the end of the shell to support the effective use of outside rows of die-holes. Close ends allow the feed material to be trapped on the face of the die. The close end roller shells assist in a great die face wear by holding back on the natural characteristics of some of the feed formulations; it works to the grooves of each side of the die face^16^. The close-end shells are available in different depths and widths and suit many applications but are generally used with poultry feed manufacturing for a well-conditioned, high-grain ration.
3. *Helical closed-end corrugated shells* they have two helices and can be used as a pair by creating opposition using one helix. Because of the helix design, the feed material tends to be pushed to the outer edges across the die face that way is helpful for dies that are experiencing problems of feed disruption of the die face. Corrugations pass throughout the contour in the form of a curve of the roller shell face. Thus, ensuring that corrugations are in proximity to the die face and leading to a smoother operation than in the case of using straight corrugated shells^17^.
4. *Dimpled shells* they have good wear characteristics because of their increased surface area. They are available with deep or shallow dimples based on the used application.
5. *Tungsten carbide shells* they are used for extremely abrasive applications when no other type of shells could handle them and would wear out quickly. They pose greater wear resistance and longer life. They are used for pellet materials that either contain ground crops like sand and dirt or with a high mineral such as cattle feed materials. Greater care should be considered when adjusting the rollers with the shell fitted^16^.

#### Analysis of variance

A factorial experiment is an experiment, which extracts information on several design factors more efficiently than can be done by the classical approach. A classical experiment uses one varied factor while the others are held constant^17^. When several sources of variation are acting simultaneously on a set of observations, the variance of the observations is the sum of the variances of the independent sources. This property makes the application of the analysis of variance particularly useful in factorial experiments. By this method, the total variation within an experiment can be broken down into variations due to; each main factor, interacting factors, and residual error. The significance of each variation is then tested. An ANOVA test is a way to find out if experiment results are significant or not. In other words, they help us to figure out if needed to reject the null hypothesis or accept the alternate hypothesis. The groups are tested to see if there is a difference between them. Groups or levels are different groups within the same independent variable^18^. Multiple research studies focus on studying the different parameters and their effect of each other on the overall quality of the pellet. The best productivity is achieved by optimizing the operating parameters within the factory through experimentation.

## Method

**Figure 2:**
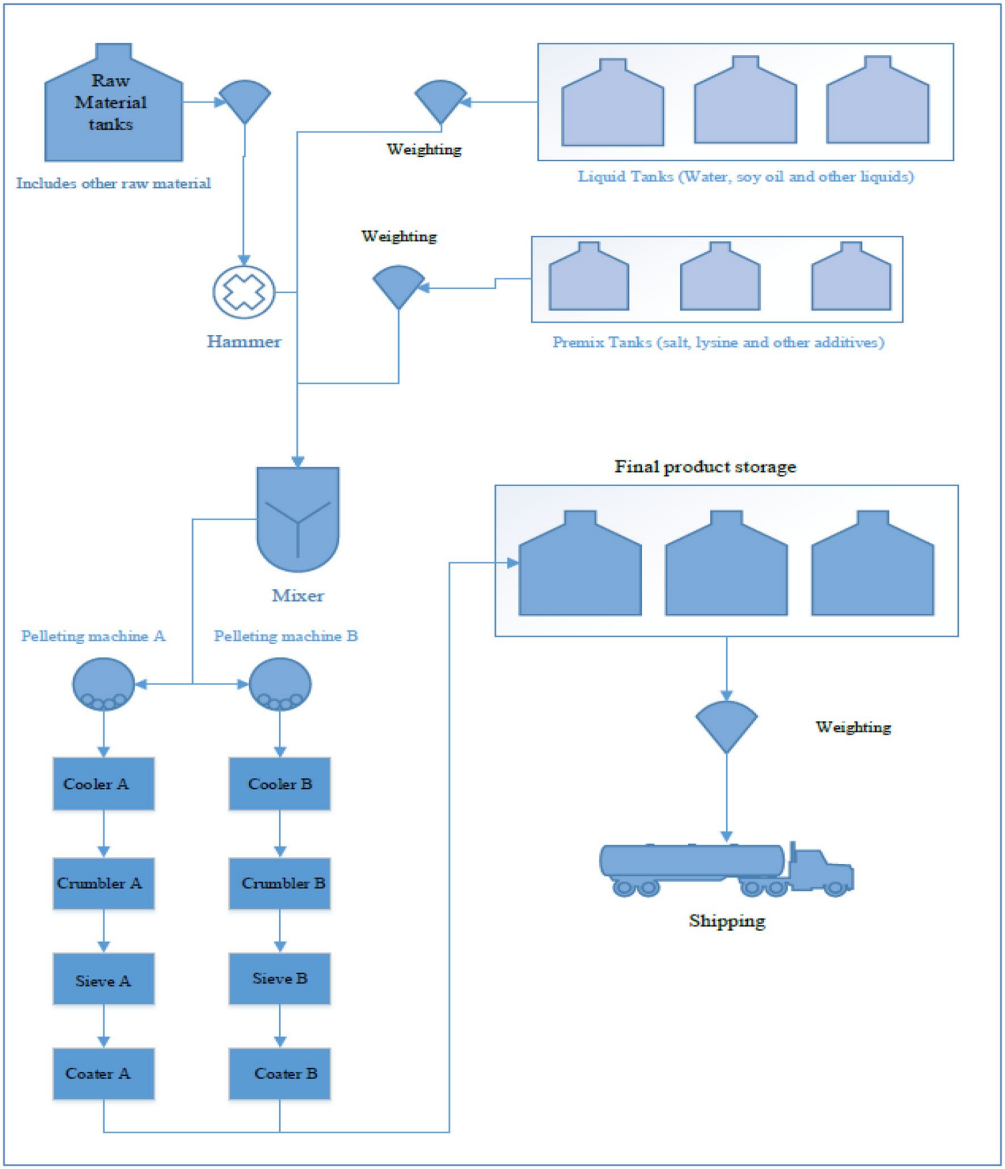
Production line flowchart.

**Figure 3:**
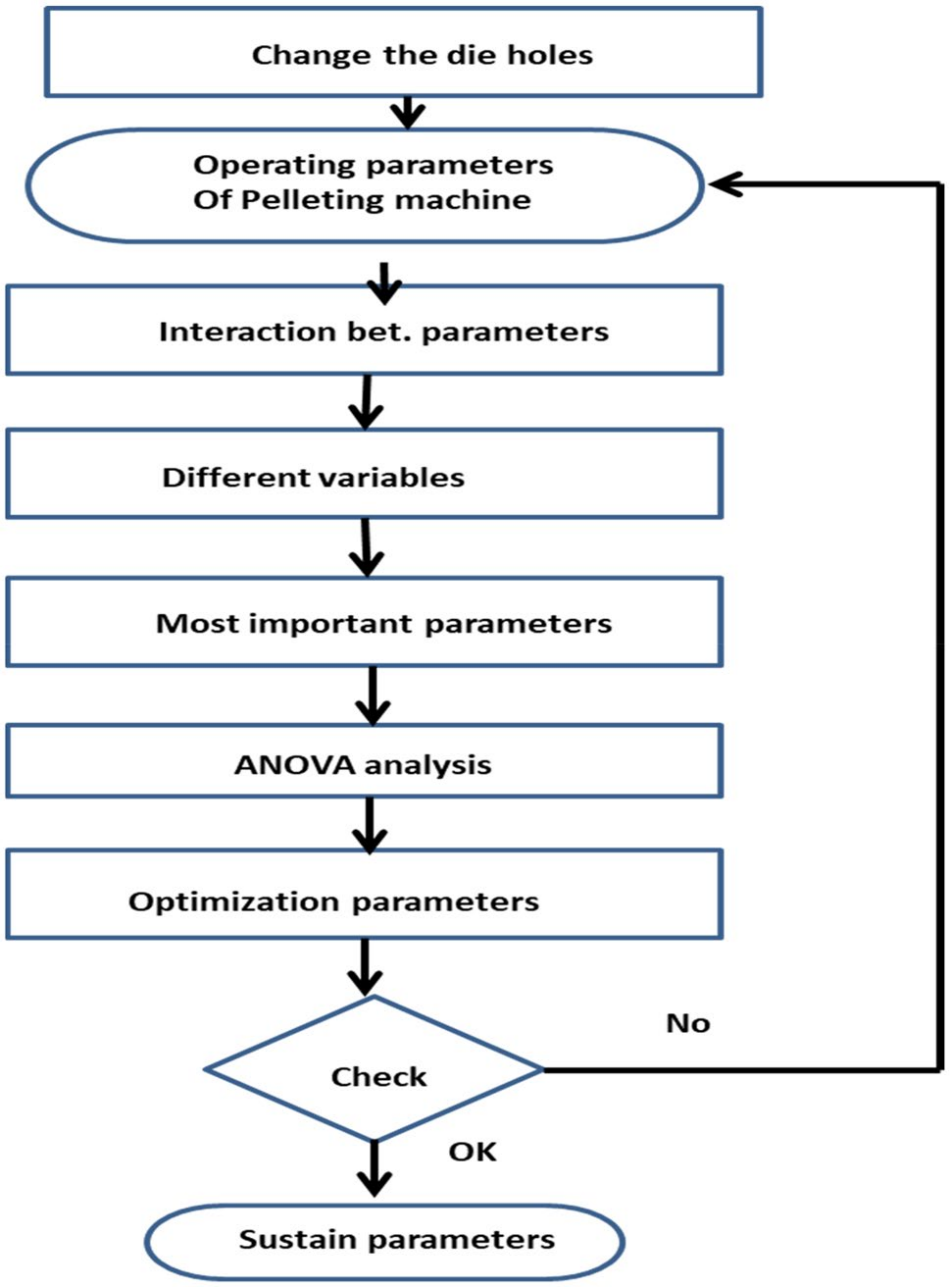
Exprimental work methodology flowchart.

Each specific organization has its exclusive constraints and problems that hinder achieving the required productivity. Analyzing all given data and parameters to find the root cause of the problems and defining the bottleneck is the first step for problem-solving. This study is adopted in an Egyptian poultry feed factory. Feed manufacturing has different processes of feed manufacturing as shown in Fig. 2. This production line has a bottleneck in the pelleting machine which increases the total downtime in the production line. Defining some pelleting machine operating parameters is very important. Analyzing the effect of different parameters on the overall quantity and quality of the pellet is also important. To reach the best production rates, improvement of the parameters must take place within the factory through experimentation. The parameters to be considered for improvement; are the operating conditions (pressure and temperature), and the die hole size of 4 mm is considered to solve the jamming problems and improve productivity. The methodology flowchart of the experimental work is presented in Fig. 3.

### Data collection and analysis

#### Stoppages of the production line

The most frequent stoppage in the production line is called jamming of the pelleting machine, where the die can no longer pass the mash feed and therefore, it is clogged and no pellets are being produced. This specific jamming stoppage is representing 58% of the total stoppages. The frequency of the jamming contributed is 44 h per Month (20 working days). During this duration, the production of the machine was reduced from an average of 11.58 tons per hour to zero consequently; the average total production loss is 509.52 Tons/month.

### Experimenting with the 4 mm dies

One of the possible solutions to the jamming problem would be the change of the hole size of the dies. This factory uses 3 mm dies. According to the experimental results achieved in previous work^4–7^, using 4 mm dies hole sizes has a greater feed quality than using (8 and 6) mm hole size. Therefore it is important to study the experiment with dies having a 4 mm die hole size and compare the results and repeat the experiment with another feed type. The following data is collected from the pelleting machine it shows the different PDI and production rates for the two different die-size holes.

### Effect of different die holes size on PDI

**Figure 4:**
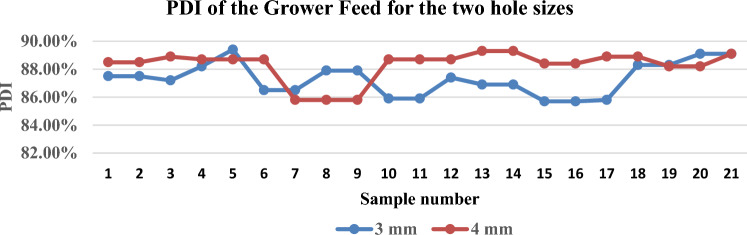
PDI of the grower feed of the two die holes size.

**Figure 5:**
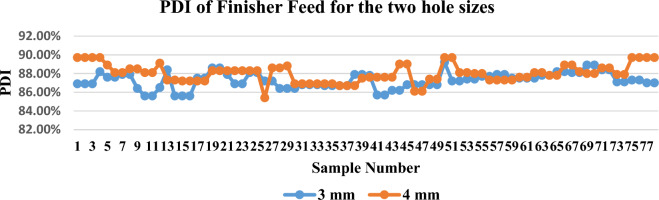
PDI of Finisher Feed for the two hole sizes.

The study was carried out on two types of feeds, the grower and the finisher. The PDI ratio is calculated for the different types of feeds for the two die-hole sizes. The obtained results showed that the PDI for the 3 mm dies hole size was 87.31% with a variance of 0.00014 while the average PDI for the 4 mm dies hole size was 88.3% with a variance of 0.00011 as shown in Fig. 4. The average of the PDI for the 3 mm dies hole size was 87.27% with a variance of $7\times {10}^{-5}$ while the average PDI for the 4 mm dies hole size was 88.01% with a variance of $9\times {10}^{-5}$ with finisher type of feed as shown in Fig. 5.

### Effect of different die holes size on productivity

**Figure 6:**
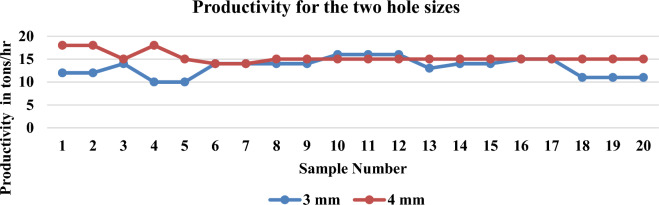
Productivity for the two hole sizes.

The collected data shows the productivity of the machine in Tons/Hr. for the two die holes sizes. The experimental study is conducted on finisher feed, as it is the main type of feed. Twenty production samples are measured randomly for the two die hole sizes during one week. The achieved results showed that the average productivity for the 3 mm dies hole size is 11.58 Tons/Hr. with a variance of 5.136 while, the average productivity for the 4 mm dies hole size is 15.35 Tons/Hr. with a variance of 1.397 as shown in Fig. 6. The achieved results also reveals that significant decrease in stoppage time in pelleting machine due to jamming from 44 to 10 h per month by using 4 mm die hole size. While the current situation looks to favor the use of 4 mm dies. A decision cannot be taken until further analysis is conducted. Therefore, some statistical tests will be conducted to ensure that there is a significant difference regarding the PDI and productivity at different die-hole sizes. One way analysis of variance is used to determine whether the die hole size is significant or not. In case it is significant, a post-ANOVA analysis will be conducted to point out the better option for both PDI and productivity.

### Analysis of variance for the PDI

Minitab software is used to determine the significance of the die-hole size for product quality. A confidence level of 95% was used for the calculations and the null hypothesis is no significant difference. The obtained results showed that the null *P*-value is less than the ‘α’ level (0.008 < 0.05), therefore, the null hypothesis is rejected shown in Table 1. Then a significant difference in the quality occurs when different die sizes are used.

**Table 1:** One way ANOVA table for grower feed.

Source	DF	Adj SS	Adj MS	F-value	*P*-value
Factor	1	0.001010	0.001010	7.93	0.008
Error	40	0.005096	0.000127		
Total	41	0.006106			

**Figure 7:**
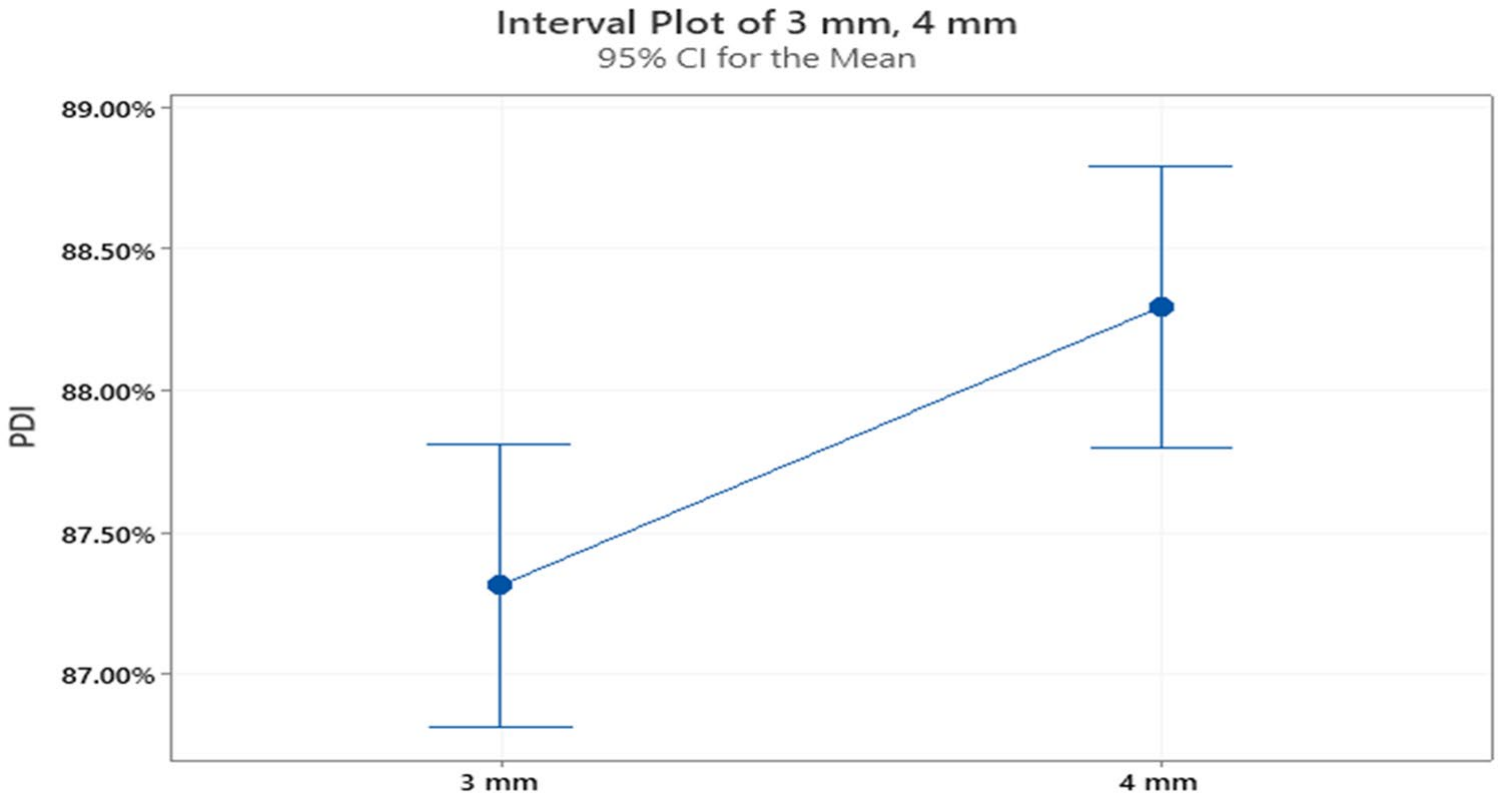
Interval plot for the PDI at the two die holes sizes for the grower feed.

It can be seen in Fig. 7 that the PDI for the 4 mm dies hole size tends to be higher with a higher mean. For the grower feed type, since a significant difference exists, it is apparent that the use of the 4 mm dies will result in a better quality product. The next study is conducted to compare the uses of the two dies hole sizes using a different type of feed. The date in the following table is for the finisher feed type.

**Figure 8:**
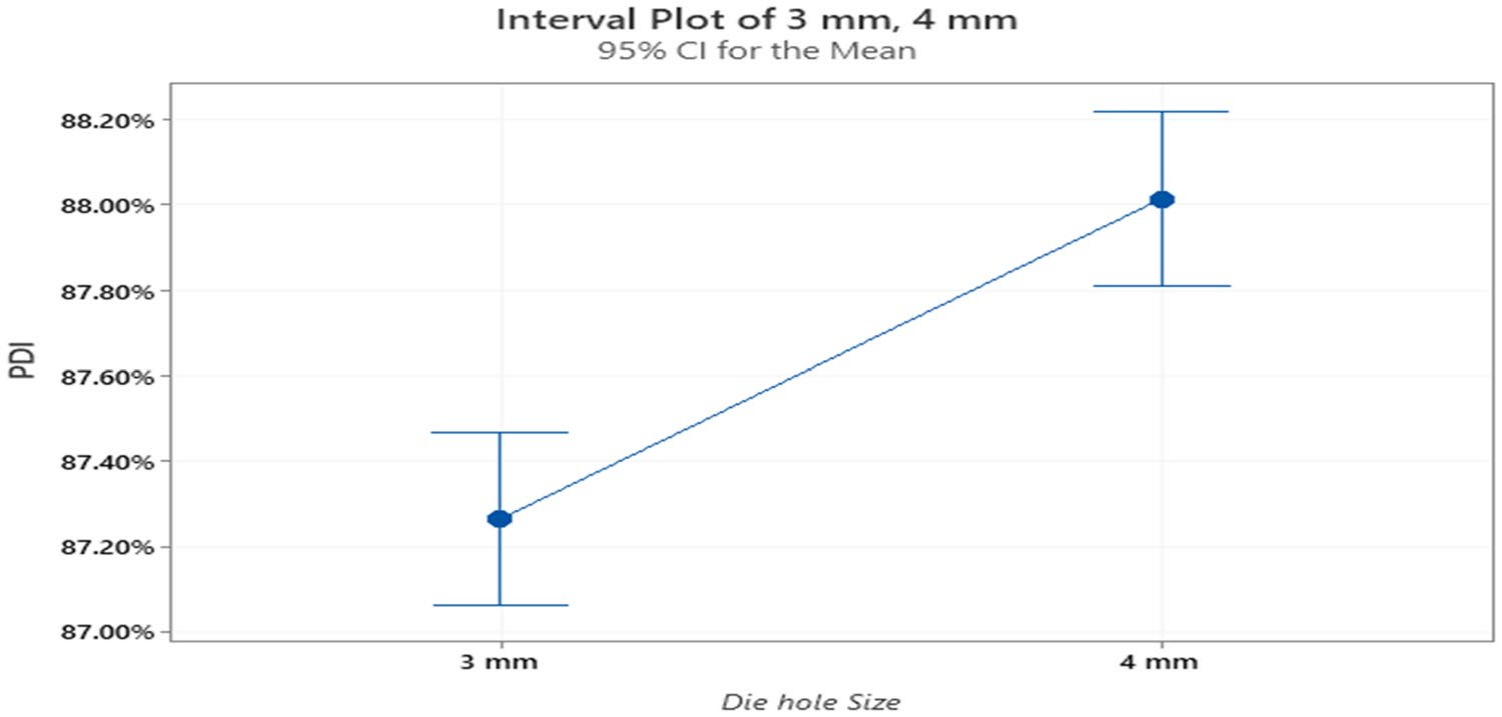
Interval plot for the PDI at the two die hole sizes for the finisher feed.

A confidence level of 95% is used for the calculations and the null hypothesis was that there is no significant difference. In this case, it is observed that the null *P*-value is less than the α level (0.000 < 0.05) as shown in Table 2. Therefore, the null hypothesis is rejected, a significant difference n the quality occurs when different dies sizes are used. It is worth mentioning that the actual *P*-value is not equal to an absolute zero. However, computer software usually rounds the actual value to zero for simplicity. It can be seen in Fig. 8 that the PDI for the 4 mm die hole size tends to be higher with a higher mean. For the finisher feed type, since a significant difference exists, it is apparent that the use of the 4 mm die will result in a better quality product.

**Table 2:** One way ANOVA table for finisher feed.

Source	DF	Adj SS	Adj MS	F-value	*P*-value
Factor	1	0.002186	0.002186	26.56	0.000
Error	154	0.012677	0.000082		
Total	155	0.014863

### Analysis of variance for the productivity

**Figure 9:**
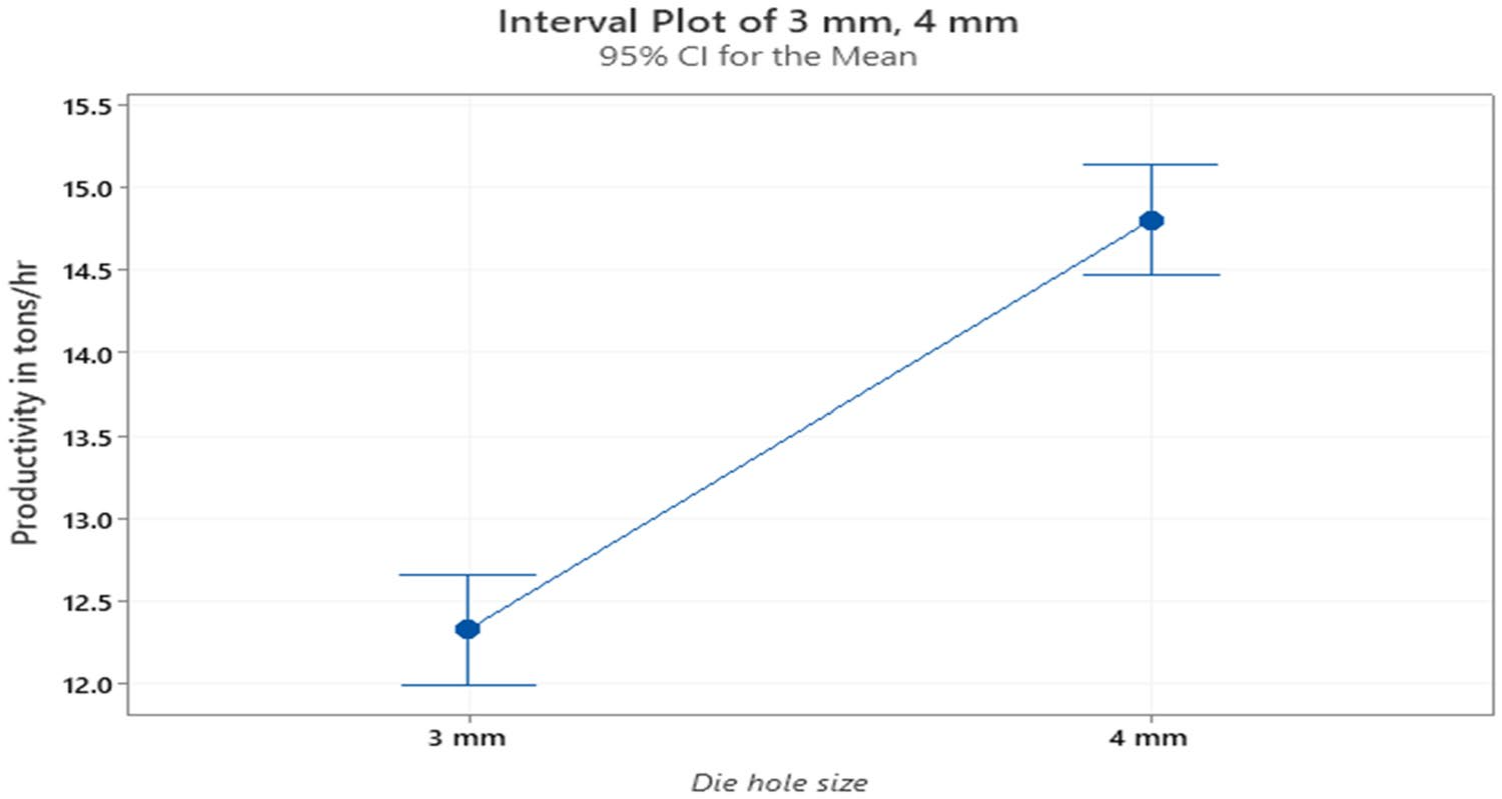
Productivity for the two die hole sizes.

The next step is to compare the productivity for the two die hole sizes to study the significance of the die hole size. The study was conducted only on the finisher type. The following table is the ANOVA table for the productivity at the two die hole sizes for the finisher feed as a main type of feed. A confidence level of 95% is used for the calculations and the null hypothesis was that there is no significant difference. In this case, it is observed that the null *P*-value is less than the α level (0.000 < 0.05). Therefore, the null hypothesis was rejected, a significant difference in productivity occurs when different die sizes are used. Once more, it is worth mentioning that the actual *P*-value is not equal to an absolute zero as shown in Table 3. However, computer software usually rounds the actual value to zero for simplicity. It can be seen in Fig. 9 that the use of the 4 mm dies results in larger productivity with a higher mean for the finisher feed type.

**Table 3:** ANOVA table for the productivity of finisher feed at the two die hole sizes .

Source	DF	Adj SS	Adj MS	F-value	*P*-value
Factor	1	228.2	228.167	107.32	0.000
Error	148	314.7	2.126		
Total	149	542.8

### Study the operating parameters

A proposed solution for the limited productivity of the factory is to study some operating parameters (pressure and temperature) and find the optimum level for each parameter. The proposed operating parameters for the experiments are taken according to the recommendation of the latest optimizing operating parameters by the quality manager. Then these parameters would be standardized. The experiments are carried out on the pelleting machine. A machine was checked by the factory to ensure that it is in suitable conditions for the experiments to take place. The productivity will be measured at different levels of these two parameters. Two-way ANOVA will then be conducted to determine the significance of each parameter. Finally, the software will be used to select the optimum operation parameters. The data was collected through many trials. The number of replications for each level is three replications after removing the failed trials or interrupted trials. There are four levels of the pressure parameter; 1.5, 1.7, 2, and 2.2 bar. The temperature consisted of three levels; these levels are intervals rather than individual values. These intervals are 75–77, 78–79, and 80–81 °C. The selected levels of the parameters are going to show the trend of what levels of each parameter are best. The obtained results from the experiments by using the ANOVA test to identify the significance of each parameter and the significance of the interaction between the two parameters are presented in Table 4. These observations can be used in the optimization process later where the best parameters will be identified to be later on standardized. Each of the interactions between the two parameters has 3 replications. These observations are entered into Minitab software so the ANOVA calculations and the suitability of the model to this case can be measured. Minitab was used to understand the significance of the parameters and the obtained results are shown in Table 5.

**Table 4:** observations from the conducted experiments.

Observations (Tons/hr.)		Pressure (bar)			
		1.5	1.7	2	2.2
Temperature (°C)	75–77	(12.7)	(10)	(14.9)	(16)
		(12.7)	(10.2)	(15)	(16)
		(12.8)	(10)	(14.8)	(16)
	78–79	(12.28)	(10.7)	(15.6)	(14)
		(12)	(10.5)	(15.32)	(15)
		(12.2)	(10.7)	(15.6)	(15)
	80–81	(12)	(11.2)	(14.9)	(13.7)
		(12)	(11)	(15)	(13.5)
		(12)	(11)	(14.8)	(13.4)

**Table 5:** ANOVA calculations on results of obtained results.

Source	DF	Adj SS	Adj MS	F-value	*P*-value
Pressure	3	122.400	40.7999	1048.54	0.000
Temperature	2	1.882	0.9411	24.19	0.000
Pressure*temperature	6	10.402	1.7336	44.55	0.000
Error	24	0.934	0.0389		
Total	35	135.617			

A confidence level of 95% was used for the calculations and the null hypothesis was that there is no significant difference. In this case, it is observed that the null *P*-value is less than the ‘α’ level (0.000 < 0.05) for the two parameters and the interaction between them. Therefore the null hypothesis that no significant difference was rejected and the two parameters and the interaction between them are significant. The results obtained from these calculations and future analysis conducted on this data can be used with confidence as a test was run to determine the fit of the model to the data and the most important results are presented in Table 6.

**Table 6:** Summary of the model results.

S	R-sq	R-sq. (adjusted)	R-sq(predicted)
0.197259	99.31%	99.00%	98.45%

The interpretation of the results is shown in Table 6, the value of S is used to test how the model responds to the data used. It is measured in the units of the response variable and represents how far the data values are from the fitted values. The lower the value of S, the better the model describes the response. R-sq is the percentage of variation in the response calculated by the model. The higher the R-sq value, the better the model fits the data. R-sq adjusted is used when it is desired to compare models that have different numbers of predictors. R-sq predicted is used to determine how well the model would predict the response for new observations. The model responds well to the data and therefore, it can be concluded with a confidence level of 95% that the temperature, pressure, and the interaction between both of them have a significant effect on productivity. With the results of the data collection and analysis phase in consideration, the implementation and results phase can be started.

### Ethics approval and consent to participate

We obtained ethical approval from the WADI poultry feed factory.

## Results and discussion

### Optimal operating parameters

**Figure 10:**
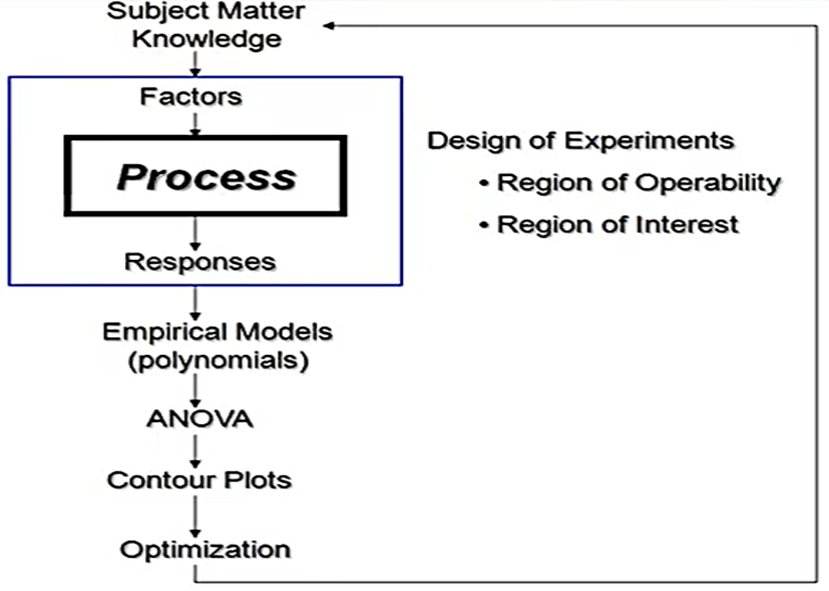
Process sequence of response surface methodology.

Design Expert is chosen as the software to analyze these experiments to obtain the optimal parameters for improvement. Design Expert is likely used in production optimization problems and it is the best use with response surface methodology (RSM). Firstly, determine which factor effects should be examined, identify the responses that need to be measured, and how many runs are needed to satisfy the objective. In statistics, response surface methodology (RSM) explores the relationships between several explanatory variables and one or more response variables. The main idea of RSM is to use a sequence of designed experiments to obtain an optimal response. This model is only an approximation but can be used because it is such an easy model to estimate and apply, even when little is known about the process. RSM can be used to maximize production by optimization of operational factors. As shown in Fig. 10.

The response surface methodology process is summarized in the following sequence:

- **Identify Responses**

After clarifying the objective, the next step is to figure out which responses to measure and how to measure them. The responses in this study were chosen to be productivity and pellet durability index (PDI) as seen in Table 7.

b) **Identify Factors and Levels**

**Table 7:** Response information.

Response	Name	Units	Observations	Analysis	Min	Max	Mean	Std. Dev	Ratio	Transform	Model
R1	Productivity	Ton/hr	36.00	Polynomial	10	16	13.19	1.97	1.60	None	cubic
R2	PDI	%	36.00	Polynomial	0.854	0.897	0.8802	0.0103	1.05	None	2FI

Factors are the inputs to the process. Factors in the experiment are controlled and set to levels prescribed by the design. In this, case the levels for temperature range from (75^◦^C to 81^◦^C) and pressure range from (1.5 to 2.2) bar as shown in Table 8.

**Table 8:** Factors information.

Factors	Name	Units	Type	Min.	Max.	Coded low	Coded high	Mean	Std. dev
A	Temperature	C	Numeric	75	81.00	−1 to 75.00	+1 to 81.00	78.39	1.98
B	Pressure	bar	Numeric	1.50	2.20	−1 to 1.50	+1 to 2.20	1.85	0.2731

The analysis can proceed once the response data has been entered. Each response must be analyzed individually, and analysis of variance is used to test the model as whole and individual terms in the model.

Other statistical tests are computed if they are applicable along with descriptive statistics to aid with verifying the correct model has been chosen and Inspect various diagnostic plots to statistically validate the model.

c) **Analysis for productivity:**

Analysis of variance showed that the *P*-value of the model is less than 0.0500 which indicates that the model terms are significant. Also, the analysis showed that the *P*-value of the lack of fit is less than 0.0500 which indicates that the lack of fit is significant as shown in Table 9.

**Table 9:** ANOVA for Response 1: Productivity.

Source	Sum of squares	df	Mean square	F-value	*P*-value	
Model	132.51	9	14.72	118.56	< 0.0001	Significant
A-temperature	0.3211	1	0.3211	2.59	0.1199	
B-pressure	75.75	1	75.75	609.99	< 0.0001	
AB	1.85	1	1.85	14.89	0.0007	
A^2^	0.0781	1	0.0781	0.6292	0.4348	
B^2^	6.33	1	6.33	51.00	< 0.0001	
A^2^B	0.0844	1	0.0844	0.6795	0.4173	
AB^2^	5.43	1	5.43	43.75	< 0.0001	
A^3^	0.0559	1	0.0559	0.4501	0.5082	
B^3^	44.80	1	44.80	360.74	< 0.0001	
Residual	3.23	26	0.1242			
Lack of fit	3.12	18	0.1733	12.70	0.0005	Significant
Pure error	0.1092	8	0.0136			
Cor total	135.74	35				

Table 10 shows the confidence intervals around the estimated cubic model coefficients. The coefficients are adjustments around that average based on the factor settings. VIFs coefficients are greater than 1 but within an acceptable range and it is less than 10 so there is no large indication for multi-collinearity, and the higher the VIF the more severe the correlation of factors. The larger the standard error of a regression coefficient, the less likely it is that this coefficient will be statistically significant.

d) **Normal Probability for productivity**

**Table 10:** Coefficients in terms of coded factors for productivity.

Factor	Coefficient estimated	df	Standard Error	95% CI Low	95% CI High	VIF
Intercept	12.87	1	0.4698	11.91	13.83	
A-temperature	− 0.3201	1	0.3442	− 1.02	0.3829	1.01
B-pressure	1.93	1	0.2953	1.32	2.53	1.04
AB	− 0.4978	1	0.4466	− 1.41	0.4143	1.04
A^2^	− 0.3260	1	0.5881	− 1.53	0.8750	1.01
B^2^	0.8505	1	0.5459	− 0.2644	1.97	1.00

**Figure 11:**
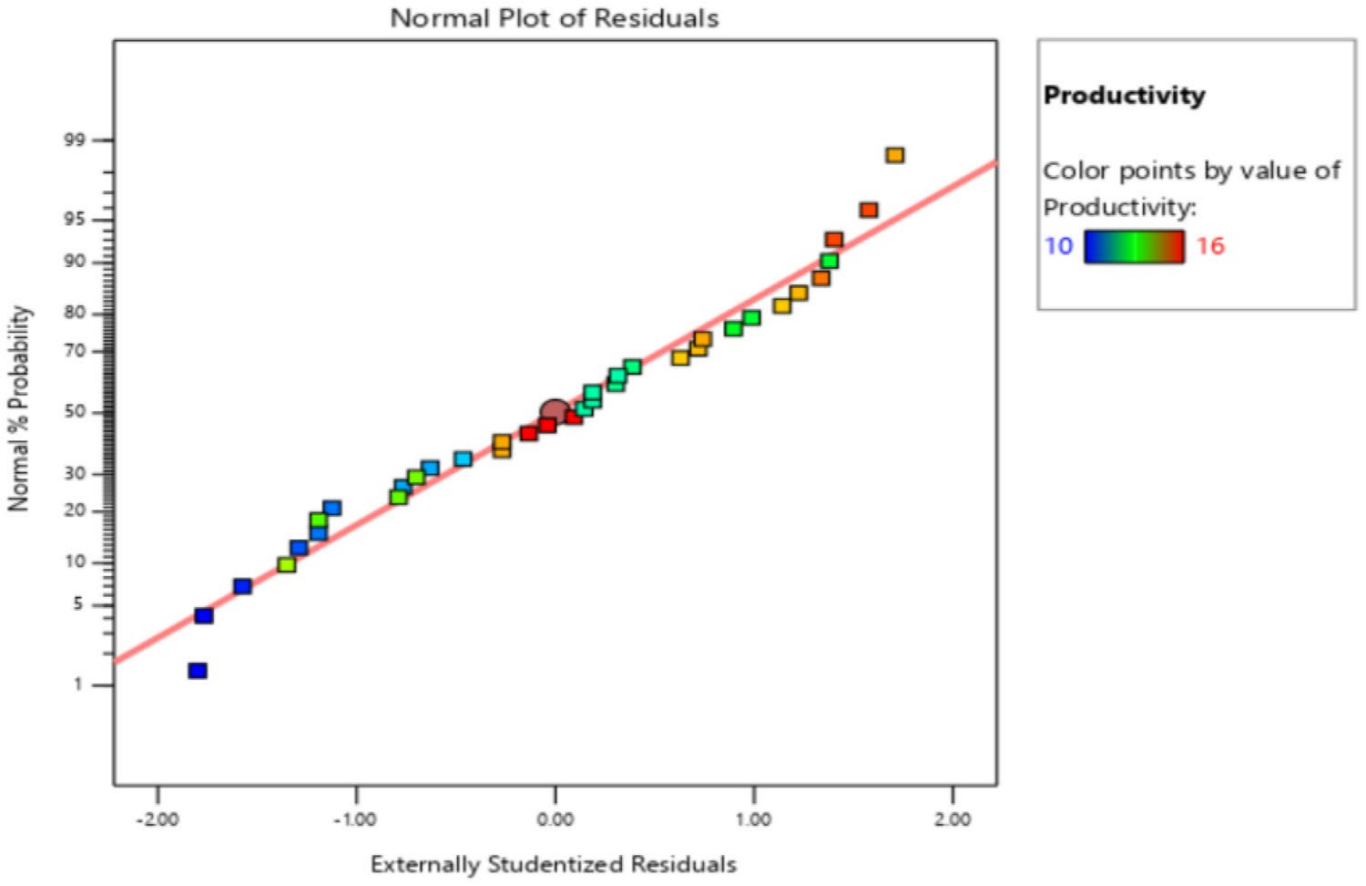
Normal probability plot for productivity.

**Figure 12:**
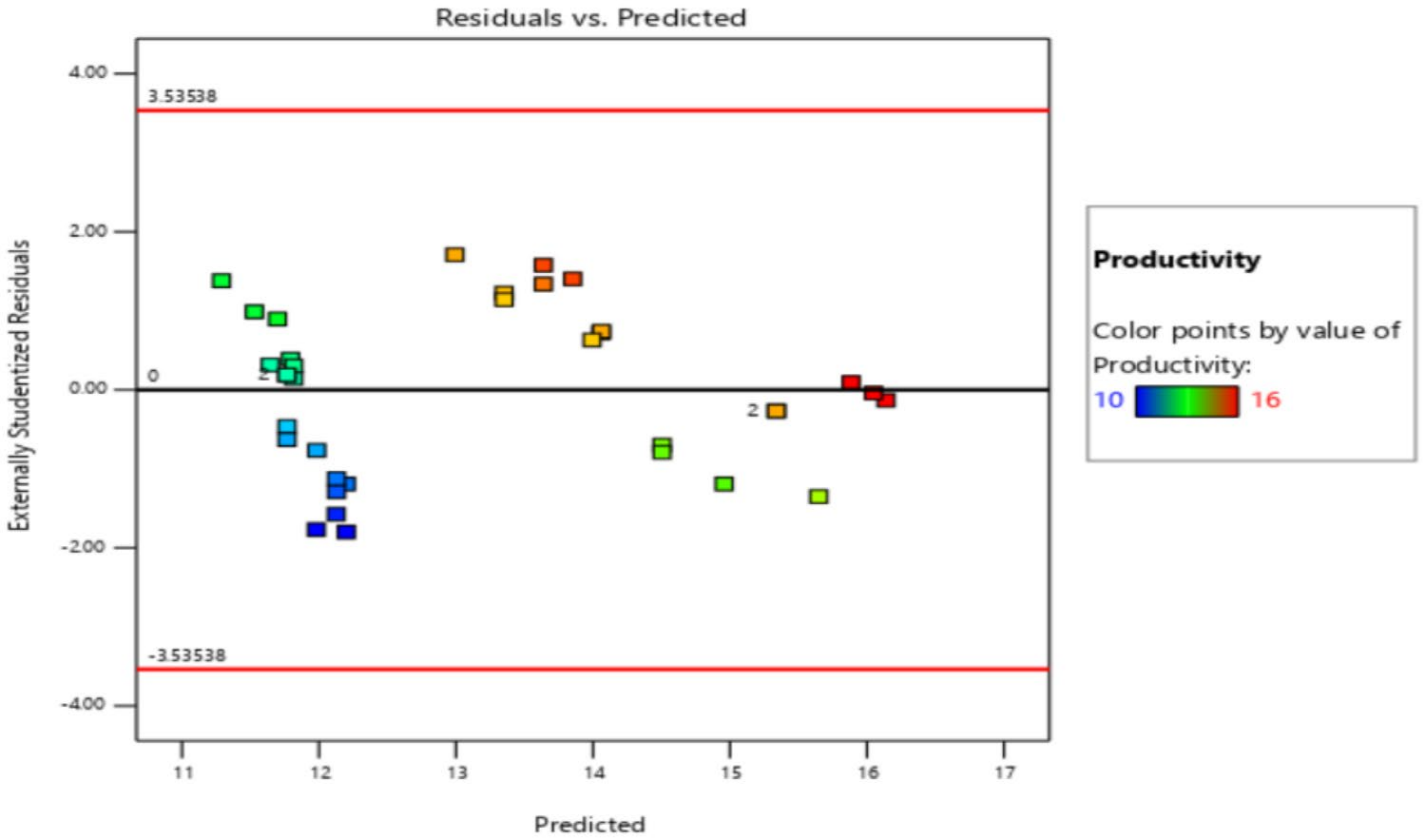
Residuals versus Predicted plot for productivity.

The residuals fall within the straight line of the normal probability plot which indicates a normal distribution as shown in Fig. 11. Some scatter should be expected even of normal data which indicates a better analysis with the response. Externally Studentized residuals based on a deletion method are the default due to excessive sensitivity for finding problems with the analysis. Figure 12 plots the residuals versus the predicted response values at constant variance. The plot consists of a random scatter (constant range of residuals across the graph). The residuals have about the same up and down spread across the plot, which indicates the analysis is reasonable.

### a) Analysis for PDI:

ANOVA analysis for PDI showed that the value of F-value is 3.03 which means that the model is significant. There is only a 4.35% chance that a large F-value could occur due to noise as shown in Table 11. *P*-values are less than 0.0500 which indicates that the model terms are significant. In this case, AB is a significant model term. *P*-values are greater than 0.100 which indicates that the model terms are not significant. If there are many insignificant model terms, the reduction of the model will improve the model.

**Table 11:** ANOVA for response 2: PDI.

Source	Sum of squares	df	Mean square	F-value	*P*-value	
Model	0.0008	3	0.0003	3.03	0.0435	Significant
A-temperature	0.0001	1	0.0001	1.31	0.2617	
B-pressure	0.0002	1	0.0002	1.71	0.1999	
AB	0.0006	1	0.0006	7.02	0.0124	
Residual	0.00029	32	0.0001			

Table 12 shows the confidence intervals around the estimated model coefficients. VIFs for PDI coefficients is greater than 1 but within an acceptable range. The coefficients are less than 10 so there is no large indication of multi-collinearity, the higher VIF the more severe the correlation of factors.

**Table 12:** Coefficients in terms of coded factors for PDI.

Factor	Coefficient estimated	df	Standard Error	95% CI Low	95% CI High	VIF
Intercept	0.8798	1	0.0016	0.8765	0.8831	
A-temperature	0.0028	1	0.0024	− 0.0022	0.0078	1.00
B-pressure	− 0.0028	1	0.0021	− 0.0070	0.0015	1.04
AB	0.0084	1	0.0032	0.0019	0.0149	1.04

**Figure 13:**
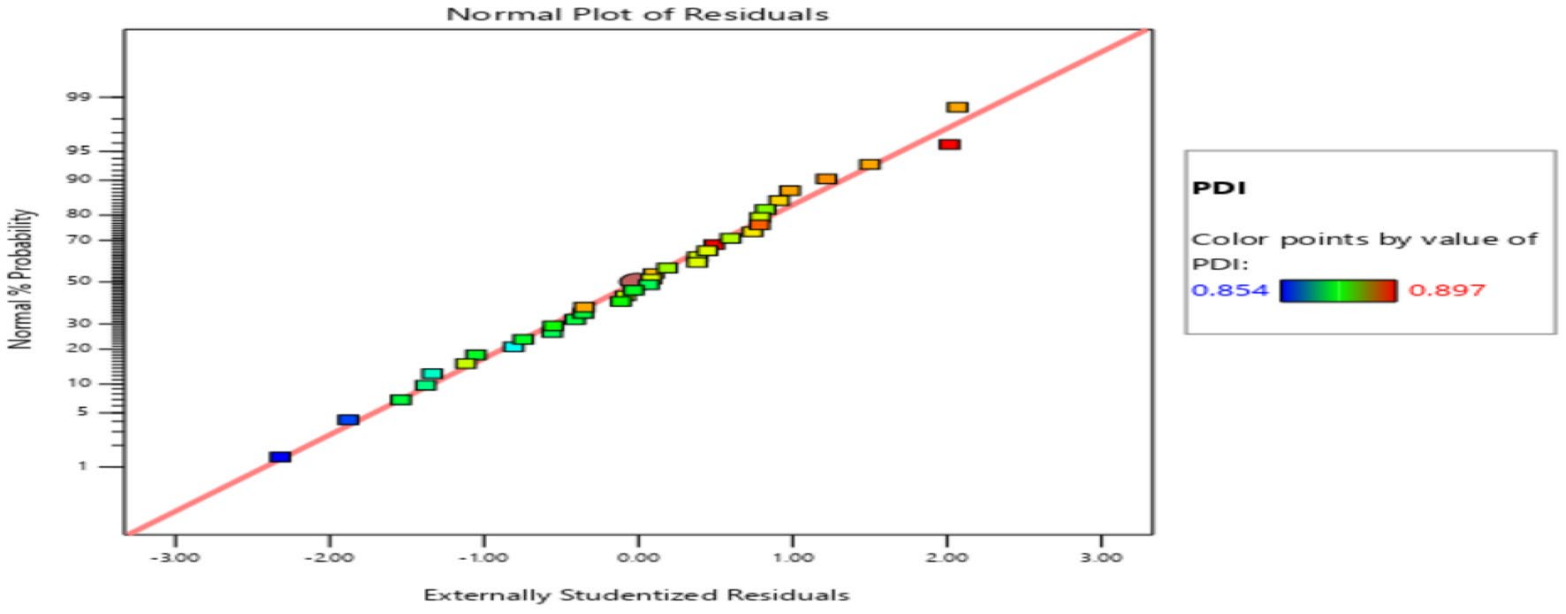
Normal probability plot for PDI.

The residuals fall within the straight line of the normal probability plot which indicates a normal distribution as shown in Fig. 13. Large deviations from a straight line suggest departures from normality, but in the figure, there is almost no deviation from the straight line which is acceptable as the ANOVA test is insensitive to even very small deviations from normality.

**Figure 14:**
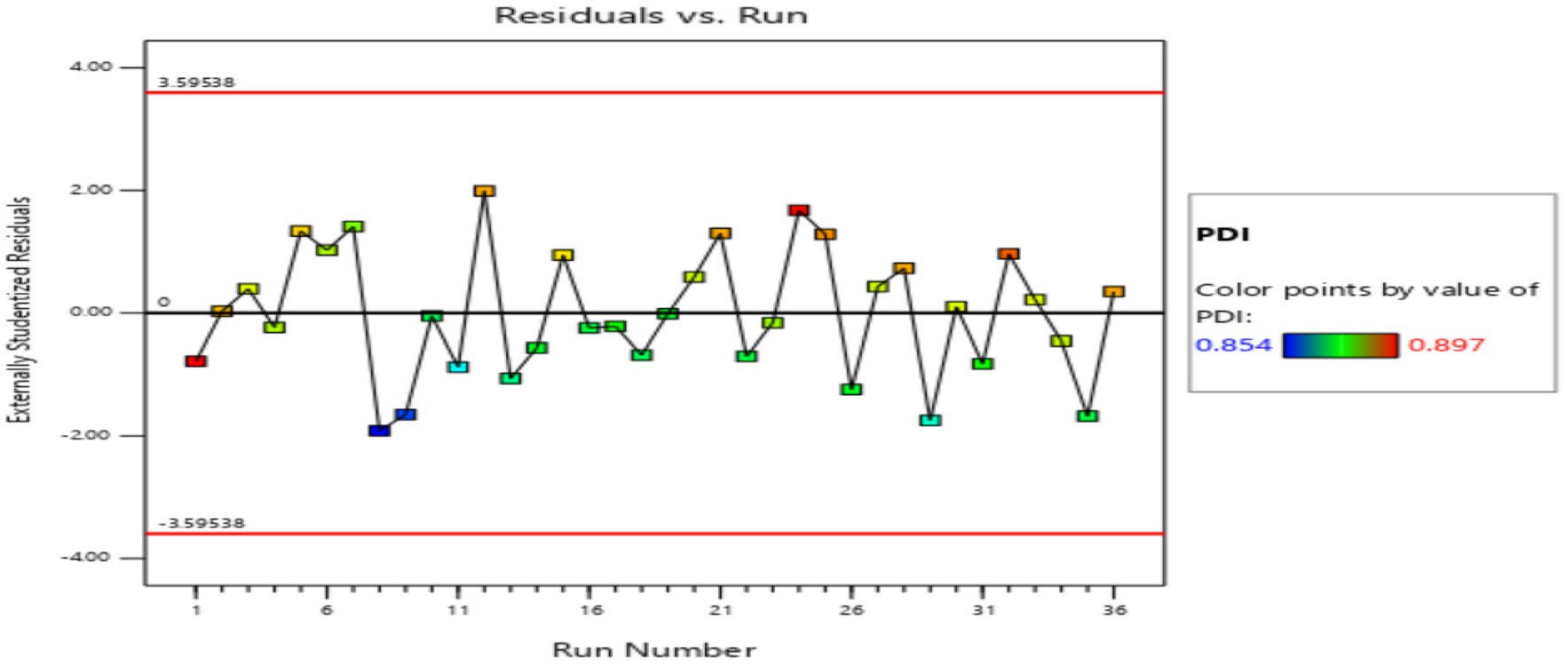
Residuals versus Run plot for PDI.

The residuals are plotted versus the experimental run order of PDI observations as shown in Fig. 14. It checks for lurked variables that may have influenced the response during the experiment. The plot represented a random scatter which indicated that a time-related variable lurking in the background. In this case, the PDI response does not suggest many variations.

### Optimization results

The optimization module searches for a combination of factor levels that simultaneously satisfy the criteria placed on each of the respond and factors. Numerical optimization uses the models to search the factor space for the best trade-offs to achieve multiple goals. Choose the desired goal for each factor and response from the menu. The possible goals are to maximize, minimize, target, within range, none (for responses only), and set to an exact value (factors only.) A weight can be assigned to each goal to adjust the shape of its particular desirability function. The importance of each goal can be changed to the other goals. Table 13 shows that the factors’ temperature and pressure goals are set as in range and their importance at 3, while responses productivity and PDI are set to maximize and their importance at 5.

**Table 13:** Optimization constraints.

Name	Goal	Lower limit	Upper limit	Lower weight	Upper weight	Importance
A:temperature	Is in range	75	81	1	1	3
B:pressure	Is in range	1.5	2.2	1	1	3
Productivity	Maximize	10	16	1	1	5
PDI	Maximize	0.854	0.897	1	1	5

As shown in Table 14, after the analysis has run a list of possible solutions is generated. The criteria for choosing the best optimal solution is based on the desirability of 1.00 the closer the solution to 1.00 the better it is. Here the best solution was at a temperature of (81 °C), with a pressure of (2.074 Bar), a productivity of (15.351 Ton/hr) and a PDI of (88.6%) which satisfy the highest productivity of the possible solutions.

**Table 14:** list of acceptable solutions.

Number	Temperature	Pressure	Productivity	PDI	Desirability	
1	**81.000**	**2.074**	**15.351**	**0.886**	**0.818**	**Selected**
2	81.000	2.077	15.343	0.886	0.818	
3	81.000	2.070	15.360	0.886	0.818	
4	81.000	2.067	15.367	0.886	0.817	
5	81.000	2.083	15.322	0.886	0.817	
6	81.000	2.062	15.372	0.886	0.817	
7	81.000	2.08	15.305	0.886	0.817	
8	75.000	1.5000	12.763	0.888	0.605	

Significant values are in bold.

**Figure 15:**
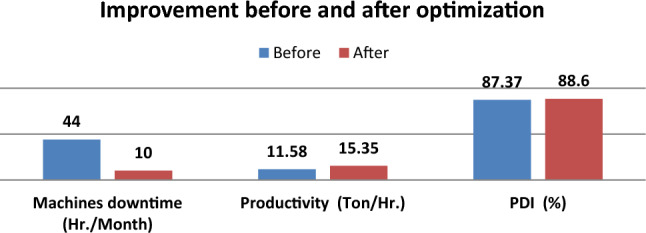
Improvement before and after optimization.

The obtained results from the statistical analysis showed that the best operation parameters are at a temperature of (81^◦^C) and pressure of (2.074 Bar) which, increase the productivity from (11.58 to 15.35) Ton/Hr. and PDI from (87.37 to 88.6) % as shown in Table 15. The average feed cost is 6000 L.E per ton. The significant results indicate that the total sales increased by 6,750,600 LE/Month as shown in Table 16. The total improvement after implementing the optimization parameters is shown in Fig. 15.

**Table 15:** Obtained results after implementation the process optimization.

Assessment criteria	Implementation of optimization parameters		Improvement
	Before	After	
Machines downtime (Hr./Month)	44	10	34
Productivity (Ton/Hr.)	11.58	15.35	3.77
PDI (%)	87.37	88.60	1.23

**Table 16:** The cost saving from improving the productivity.

Assessment criteria	Quantity saved (Ton)	Equivalent cost (LE)	Total sale cost (LE)
Decrease machines downtime (Hr./Month)	34 × 15.35 = 521.9	521.9 × 6000	3,131,400
Increase productivity (Ton/Month)	3.77 × 8 × 20 = 603.2	603.2 × 6000	3,619,200
Total saving (LE/Month)			6,750,600

## Conclusion

The proposed paper is considered a research methodology with a case-based analysis and optimize the operating parameters of the pelleting machine to improve productivity. In addition, it decreases machine downtime due to jamming which, supports in achieving the objectives of the organization. An experimental investigation for optimized operational parameters of pelleting machine for poultry feed mill production was conducted by using Minitab and Design expert software method. ANOVA was performed to evaluate the effects of the die hole size and the operating parameters on productivity and product quality. Results presented that the die hole size and the two parameters temperature and pressure have had the most significant effect on productivity and product quality. The optimal solution provided the values optimized operating parameters: 81 (°C) temperatures and 2.074 (Bar) pressure. A practical evaluation of the optimization values was performed. Results showed that the productivity increased from (11.58 to 15.35) Tons/Hr. and PDI improved from (87.37 to 88.6) %. The obtained results also indicated that the changing die size of the pelleting machine from (3 to 4) mm had a great impact on decreasing the total stoppages due to jamming from (44 to 10) Hr. per month. The stable productivity and the Pellet durability index of the production line which were designed based on the optimized operating parameters have confirmed the appropriateness of the values suggested. The total sales increased by 6,750,600 LE/Month.

## Data Availability

The datasets used and/or analyzed during the current study are available from the corresponding author on reasonable request.

## Abbreviations

PDI: Pellet durability index
NSP: Non-starch polysaccharides
ANOVA: Analysis of variance
